# Transforming research to improve therapies for trauma in the twenty-first century: an alternative perspective

**DOI:** 10.1186/s13054-024-04913-3

**Published:** 2024-04-23

**Authors:** Geoffrey P. Dobson, Jodie L. Morris, Hayley L. Letson

**Affiliations:** Heart, Sepsis and Trauma Research Laboratory, College of Medicine and Dentistry, James Cook University, 1 James Cook Drive, Townsville, QLD 4811 Australia

We read with interest the perspective of Juffermans and colleagues on transforming research to improve therapies for trauma care in the twenty-first century [1]. We would like to offer a different perspective. We think a more interesting approach is to ask how historians of science 50 or 100 years from now might view our progress in the early twenty-first century? Instead of looking into the future from where we stand today, we move the needle into the future and reflect backwards. We agree with Juffermans and colleagues that specific treatments for bleeding are largely lacking [1], however, they fail to delve deeper. Why are there so few safe and effective drugs to treat hemorrhage, traumatic brain injury or burn trauma? Why have there been so few breakthroughs? We argue the lack of drug therapies is a consequence of the treat-as-you-go symptoms-based approach, rather than a more integrated systems-based approach [2, 3]. The current practice of identifying and treating one defect at a time, and so on down the line, often leads to what US trauma surgeon William C. Shoemaker deemed: “an uncoordinated and sometimes contradictory therapeutic outcome” [4]. *In the twenty-first century, a new revolution is required to better understand how the body responds to trauma, identify new markers to detect its progression and discover new system-acting drugs to treat it*.

Candidate therapies listed in Table 3 in Juffermans’ manuscript [1] are largely single-nodal target therapies. Single-nodal or branch-point targets fail to appreciate the complexity of the system. Students of biomedicine need to appreciate that probing the underlying mechanisms of how drugs affect cells or tissue culture is only one step toward understanding how they behave inside a living organism. Adopting the “Omics” technologies to drill deeper into cellular mechanisms has occurred at the expense of whole-body systems analysis. The current single-nodal approach can be traced back to the molecular revolution of the twentieth century, which began in earnest around 1953 after the discovery of DNA [3, 5]. Nobel Laureate Sir Francis Crick embodied this position when he wrote “the ultimate aim of the modern movement in biology is to explain all biology in terms of physics and chemistry” [6]. Indeed, this single-nodal mindset has influenced the way we study, diagnose, treat, and prevent injury and diseases [7]. The issue was anticipated over 15 years ago by the USA Food and Drug Administration (FDA) who recommended that: “strengthening and rebuilding the disciplines of physiology, pharmacology and clinical pharmacology, will be necessary to provide the capacity to develop and evaluate new biomarkers and bridge across animal and human studies” [8]. Unfortunately, there doesn’t appear to have been follow-up. *The key point is that despite an overwhelming amount of mechanistic data from basic scientific research, its relevance to the workings of the whole body has not kept pace*. Modern science has forgotten to put Humpty Dumpty back together again. Reductionism is important in breaking a complex system into its simpler parts, but it does not do away with the system. This flawed way of thinking, we believe, is a major contributor for the high failure rate of translating promising new trauma drugs in clinical trials [9]. A systems approach is much more likely to increase animal to human translation success.

A second major reason for lack of trauma drug breakthroughs is the choice of animal model and its suitability for future clinical translation. Although animal models were discussed in Juffermans’ review [1] there was no discussion on specific-pathogen free (SPF) animals. If the research goal is to examine a particular mechanism or pathway, the use of SPF animals may be the right choice. However, if the goal is to develop and translate new drugs to humans, conventionally bred and housed animals are the choice because SPF animals have altered gut microbiomes and different immune systems [8, 10]. *Conventional animals are more representative of the human condition* [8, 10]. SPF animals were introduced in the 1960s to minimize disease and infection as variables in biomedical research, however, removing a list of pathogens using selected antibiotics does not represent the ‘normal’ condition. Today, SPF status has become a variable itself in biomedical research [10]. Letson and colleagues showed SPF rats displayed abnormal hemodynamics, bleeding, arrhythmias, and hematological status in response to anesthesia and surgery compared to conventional, non-SPF, animals [10, 11]. Furthermore, a landmark study of Beura and colleagues showed that ‘standard’ SPF adult mice had “immature” immune systems and were more prone to infection compared to wild mice [12]. Co-housing SPF animals with pet store mice reversed the problem, and produced mice with immune systems closer to adult humans [12]. Similarly, Rosshart and colleagues showed SPF-type mice reconstituted with natural microbiota exhibited reduced inflammation and increased survival following influenza virus infection, and they showed improved resistance against colorectal tumorigenesis [13, 14]. Finally, as discussed by Juffermans and colleagues, there are other reasons for why so few drugs translate from animals to humans, including poor clinical trial design, patient selection, and many other factors [1].

What would a systems-based drug look like? Ideally, a systems-based treatment would initially target the early immune-driven central nervous system (CNS) stress response, promote CNS-cardiovascular coupling, protect the endothelial-glycocalyx, reduce immune dysfunction, prevent hyperinflammation, correct coagulopathy, and deliver sufficient O_2_ to the tissues to maintain mitochondrial function [3, 15]. No such drug exists. In conclusion, we agree with Juffermans and colleagues that the pathophysiology of trauma remains incomplete, however, we differ in the future approach to improve therapies. We propose there is an urgent need for a paradigm shift in drug development from a symptoms-based to a systems-based approach [2, 3]. If the single-nodal mindset and SPF animal choice continue to dominate our thinking [1], future historians may write: “most scientists of the early twenty-first century were caught up in the molecular revolution to advance medicine, and could not break free, until research bodies decided to fund and realign their focus on the whole-body system”. When this realignment might occur is unclear, however, the sooner the better to improve therapies for trauma in the twenty-first century.

## Data Availability

The datasets during and/or analysed during the current commentary can be made available from the corresponding author on reasonable request.
